# Acute Toxicity Assessment, In Vitro Antacid and Cytoprotective Effects of Root Bark Aqueous Extract of *Diospyros mespiliformis* on Water Immersion Stress-Induced Gastric Ulcers in Rats

**DOI:** 10.1155/cjgh/8936445

**Published:** 2025-06-06

**Authors:** Vandza Luc Vandi, André Perfusion Amang, Christophe Mezui, Gael Tchokomeni Siwe, Odile Baponwa, Sidiki Aboubakar, Joseph Fleurie Emakoua, Paul Vernyuy Tan

**Affiliations:** ^1^Department of Biological Sciences, Faculty of Science, University of Maroua, P.O. Box 814, Maroua, Cameroon; ^2^Department of Biological Sciences, Higher Teachers' Training College, University of Yaoundé I, P.O. Box 47, Yaoundé, Cameroon; ^3^Department of Biotechnology and Pharmacognosy, Faculty of Science, University of Ebolowa I, P.O. Box 118, Ebolowa, Cameroon; ^4^Department of Animal Biology and Physiology, Faculty of Science, University of Yaoundé I, P.O. Box 812, Yaoundé, Cameroon; ^5^Department of Biological Sciences of Living Organisms, Faculty of Science, University of Garoua, P.O. Box 346, Garoua, Cameroon

**Keywords:** acute toxicity, antacid, cytoprotective, *Diospyros mespiliformis*, gastric ulcers

## Abstract

**Objective:** The objective of the present study was to evaluate the antacid and cytoprotective effects of root bark aqueous extract of *Diospyros mespiliformis* (RBAEDM).

**Materials and Methods:** Thirty rats were grouped into six groups of five rats each, namely, three control groups (normal, negative and positive) and three test groups. These animals were treated with distilled water (normal and negative controls), verapamil (positive control) and the extract at doses of 100, 200 and 400 mg/kg (test groups). One hour after treatment, gastric ulcers were induced in all animals by immersion in water (22 ± 1°C) for 5 h except for the normal control. The ulcerated surface, mucus mass, in vivo oxidative stress parameters and nitrite levels were determined. In vitro antacid activity of the extract was evaluated on artificial gastric juice by the determination of pH, neutralization time and antacid capacity. Acute toxicity of extract was evaluated.

**Results:** Treatment with RBAEDM showed a significant (*p* < 0.01, *p* < 0.001) decrease of ulcerated surface with a percentage of inhibition between 5.58% and 60.46%. The decrease in the ulcerated surface was accompanied by a significant (*p* < 0.001) increase in mucus production at 400 mg/kg. Treatment with RBAEDM also showed a significant (*p* < 0.001) decrease in malondialdehyde (MDA) levels and a significant increase in superoxide dismutase (SOD) level, catalase (CAT) activities, in addition to nitrite levels in stomachs. In artificial gastric juice, the RBAEDM caused a significant increase (*p* < 0.001) of neutralizing time and the number of neutralized H^+^ ions compared to distilled water. No change in behavioural parameters and no death was observed after administration of the extract at 2000 mg/kg.

**Conclusion:** RBAEDM inhibited ulcer occurrence by the stimulation of mucus production, increase of antioxidant enzyme activity and NO production. Moreover, this study revealed that the studied extract could exert a strong anti-acid capacity in vitro due its ability for the neutralization of H^+^ ions. DL_50_ of RBAEDM was greater than 2000 mg/kg.

## 1. Introduction

Stomach is an organ of the gastrointestinal tract that plays an important role in food digestion [1]. Indeed, it is involved in the process of protein digestion via acid secretion, which is responsible for the activation of a proteolytic enzyme called pepsinogen [2, 3]. Excessive acid secretion by the stomach can lead to inflammation of the gastric mucosa, which causes gastric ulcers. Acid hypersecretion can result either from the stimulation of histamine, gastrin and acetylcholine receptors in the case of stress, or from uncontrolled gastrin production in the case of Zollinger–Ellison syndrome [4]. However, the body has several defence mechanisms against the deleterious action of hyperacidity on the gastric mucosa such as secretion of mucus, bicarbonate and endogenous antioxidant, as well as increased blood flow by NO production [5].

In case of imbalance between the aggression and defence factors of the gastric mucosa, the inhibition of acid secretion could provide relief or even treatment [6]. With this in mind, several drugs such as antacids are used for the treatment of gastrointestinal tract disorders related to gastric acid hypersecretion [7]. For many years, antacids have been widely used in ulcer therapy [8, 9]. According to WHO (2017), more than USD10 billion is spent on antacid utilization worldwide each year. Oral administration of antacids provides instant and rapid relief to patients [3]. However, side effects and drug interactions are the main challenges associated with the use of synthetic antacids [8]. In response to such challenges, people are resorting to herbal medicines as an alternative.

*Diospyros mespiliformis* is a wild fruit tree of Ebenaceae family confined to tropical and subtropical regions widely used in ethnomedicine for the treatment of several diseases including gastric ulcers [10, 11]. Several pharmacological studies have shown its analgesic, anti-inflammatory, antipyretic, sedative and antimalarial properties [12, 13]. Previous works have shown the gastro-protective effects of the aqueous extract of this plant on three models of gastric ulcer induction namely, HCl/ethanol, HCl/ethanol pre-treated with indomethacin and indomethacin [14]. Other work has shown the antisecretory potential of the extract of this plant [15]. However, its ability to neutralize gastric acidity and prevent gastric ulcer formation resulting from stress exposure has not yet been studied. Hence, the aim of the present study was to evaluate the antacid and cytoprotective effects of the RBAEDM on stress-induced gastric ulcers by water immersion in rats.

## 2. Materials and Methods

### 2.1. Animal Material

Male albinos Wistar rats aged 12 ± 2 weeks old and weighting 180 ± 20 g were used in this study. All the animals used in the study were raised at the Animal Physiology and Pharmacognosy Laboratory of the University of Maroua. Prior authorization for the use of laboratory animals in this study was obtained from the Cameroon National Ethics Committee (registration number: FWA-IRB00001954). Otherwise, the use, handling and care of animals were done in adherence to the European Convention for the Protection of Vertebrate Animals Used for Experimental and Other Purposes (ETS-123), with particular attention to Part III, articles 7–9 [16].

### 2.2. Plant Material

The root bark of *Diospyros mespiliformis* plant was harvested in the Mokolo locality, Far-North region of Cameroon (N10°44′12.93072″; E13°47′3.74784″). The plant identification and authentication were performed by the experts at the Herbarium of the Garoua School of Fauna where it was registered under the voucher number HEFG/01404. After harvesting the root barks of *D. mespiliformis* plant, they were dried in the shade and ground into a powder form for the preparation of the extract.

### 2.3. Preparation of the RBAEDM

Three hundred grams (300 g) of powder were macerated in 3 L of distilled water for 24 h. After filtration using Whatman paper No. 3, the solution was kept in an oven at 50°C to facilitate evaporation until we obtain 12 g of extract (4% yield).

### 2.4. Gastric Lesions Induced With Cold Stress Water Immersion

Gastric ulcers were induced with water immersion stress in rats using the method described by Tagaki and Okabe [17]. After 48 h of non-hydric fasting, 30 rats were grouped into six groups of five rats each including three test groups treated with the extract at doses of 100, 200 and 400 mg/kg, and three control groups treated with distilled water (1 mL/200 g) for normal and negative controls, and verapamil (50 mg/kg) for the positive control. One hour later, all animals except normal control were placed in small individual cages and immersed in water at 21°C–23°C up to the xiphoid. Five hours (5 h) later, the animals were sacrificed under diazepam/ketamine anaesthesia. Stomachs were collected for measurement of gastric lesions and mucus mass. These stomach samples were also used for antioxidant and nitrite assays.

#### 2.4.1. Ulcerated Surface and Ulcer Index

Ulcerated surface and ulcer index were calculated as described by Tan et al. [18]. Ulcerated surface: length x width. The scores are allotted as follows: no ulcer = 0.0; ulcer surface less than or equal to 0.5 mm^2^ = 1; ulcer surface greater than 0.5 and less or equal to 2.5 mm^2^ = 2; ulcer surface greater than 2.5 and less or equal to 5 mm^2^ = 3; ulcer surface greater than 5 and less or equal to 10 mm^2^ = 4; ulcer surface greater than 10 and less or equal to 15 mm^2^ = 5; ulcer surface greater than 15 and less or equal to 20 mm^2^ = 6; ulcer surface greater than 20 and less or equal to 25 mm^2^ = 7; ulcer surface greater than 25 and less or equal to 30 mm^2^ = 8; ulcer surface greater than 30 and less or equal to 35 mm^2^ = 9; ulcer surface greater than 35 mm^2^ = 10. The ulcer index was calculated with the following formula:

(1) $$\begin{array}{c} \text{UI}=\frac{1}{n}\overset{n}{\underset{1}{\sum }}\text{scores}\pm \text{Standard Error on Mean}. \end{array}$$

#### 2.4.2. Percentage of Inhibition of Gastric Ulcer

Percentage of inhibition is the preventive value of all antiulcer agents used against ulceration severity. This was determined with the negative control using the following formula:

(2) $$\begin{array}{c} \%I=\frac{\text{Ulcer index of negative control}-\text{ulcer index of test}}{\text{Ulcer index of negative control}}\times 100. \end{array}$$

### 2.5. Determination of RBAEDM Antacid Capacity

#### 2.5.1. Preparation of Artificial Gastric Juice

Two grams (2 g) of NaCl and 3.2 mg of pepsin were dissolved in 493 mL of distilled water. Seven (7 mL) of 12M hydrochloric acid was added to 493 mL of distilled water, resulting in 1000 mL of artificial gastric juice. The pH of the gastric acid solution was adjusted to 1.2.

#### 2.5.2. Determination of the Temperature Effect on pH

Ninety millilitres (90 mL) of the prepared extract solution or water or sodium bicarbonate was added to 100 mL of artificial gastric juice at pH 1.2. The pH was measured after the addition of the different substances to the artificial gastric acid using the pH meter at 25°C and 37°C. This experiment was repeated five times for each solution.

#### 2.5.3. Determination of the Neutralizing Time of the RBAEDM

The artificial stomach model of [19] with some modifications was used. 90 mL of samples (water, extract at different concentrations or sodium bicarbonate) were added to 100 mL of artificial gastric juice at pH 1.2 and maintained at 37°C. The artificial gastric juice was infused at a rate of 3 mL per minute (3 mL/min), and an equal amount of the reaction mixture was drained at the same time. The pH meter was immersed in the solution to monitor the changes in pH. The neutralizing time was noted when the pH returned to its initial value of 1.2.

#### 2.5.4. Determination of the In Vitro Neutralizing Capacity of the Extract

The model of [20] using an in vitro titration with some modifications was employed. The solutions were titrated with artificial gastric juice with the aim of reaching a pH of 3. The in vitro neutralizing capacity of the extract was determined by adding artificial gastric acid to the freshly prepared solution of the extract (90 mL) contained in 250-mL beaker. The consumed volume of artificial gastric juice was measured. The total acid (H^+^) consumed was calculated using the formula H^+^ (mmol) consumed = 0.063096 mmol/mL × volume (mL).

### 2.6. In Vivo Antioxidant Activity of RBAEDM

In vivo antioxidant capacity of the extract was evaluated in stomach homogenates. The determination of total protein was done according to the method of Biuret [21] and MDA level according to the protocol of [22]. The activity of superoxide dismutase (SOD) [23], catalase (CAT) [24] and reduced glutathione (GSH) [25] was determined.

### 2.7. Quantification of Nitrite Level

The nitrite in the stomach homogenates was measured with the Griess reagent according to the method described by [26]. The chromophore absorption during nitrite deionization with sulphanilamide coupled to naphthalenediamine (NED) was read at 546 nm. The product obtained was proportional to the amount of nitrite present in the sample. The nitric oxide level was determined from the calibration curve established from different concentrations of NaNO_2_.

### 2.8. Study of the Toxicity of *Diospyros mespiliformis* Extract

The acute oral toxicity of the RBAEDM was assessed in rats using the protocol of Organisation for Economic Cooperation and Development (OECD), guideline no. 423 for the testing of chemical substances (acute toxicity class method).

Six female rats were divided into two groups of three rats each. The rats in the control group were given distilled water (10 mL/kg), while the first test group received the extract at 2000 mg/kg by single-dose gavage. The same methods and doses were repeated 48 h later on six animals (three for the control and three for the test), which were the confirmation groups.

The treated animals were deprived of food and water for the first 4 h postgavage. The animals' clinical parameters were observed for the first 30 min after treatment, then periodically (with particular attention paid to the first 4 h) for 24, 48 h and daily for 14 days. Parameters such as mortality and mobility, behaviour, aggressiveness, pain sensitivity, salivation as well as body weight, water consumption and mortality were assessed [27].

### 2.9. Statistical Analysis

The data were analysed using GraphPad Prism 5.03 software. Statistical analysis was performed by one-way analysis of variance (ANOVA) followed by Newman–Keuls post-test. The values were expressed as mean ± standard error of the mean (SEM). *p* values < 0.05 were considered as statistically significant.

## 3. Results and Discussion

### 3.1. Effect of RBAEDM on Water Stress Immersion-Induced Gastric Ulcers

#### 3.1.1. Macroscopic Observation of Water Immersion Stress Ulcerated Stomachs

**Figure 1:**
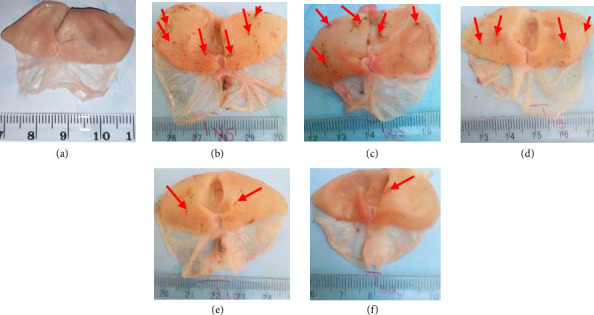
Photograph of stomachs ulcerated by cold stress water immersion. (a) Normal control; (b) negative control; (c) 50 mg/kg of verapamil; (d) 100 mg/kg of RBAEDM; (e) 200 mg/kg of RBAEDM; and (f) 400 mg/kg of RBAEDM; ⟶: indications of gastric lesions.

No lesions were observed in the normal group (Figure 1(a)). Water immersion-stressed rats showed lesions at their glandular region. These lesions were more intensified in number for the negative and positive controls (Figures 1(b) and 1(c)), then decreased in a dose-dependent manner (100, 200 and 400 mg/kg) in animals treated with RBAEDM (Figures 1(d), 1(e), and 1(f)) (Figure 1).

#### 3.1.2. Cytoprotective Effects of RBAEDM on Water Immersion Stress-Induced Gastric Ulcers

The effect of RBAEDM on the ulcerated surface, ulcer index, percentage inhibition and mucus mass is recorded in Table 1. The table reveals that the extract administration at doses of 100, 200 and 400 mg/kg resulted in a decrease in the ulcerated surface of 28.40, 15.00 and 9.20 mm^2^, respectively, meaning that the decreasing effect was dose-dependent. With respect to the negative control (37.60 mm^2^), the decreasing effects correspond to an ulcer inhibition of 5.58%, 32.55% and 60.46%, respectively. The administration of the extract at 400 mg/kg and verapamil (50 mg/kg) significantly (*p* < 0.001) increased mucus production (70.64 and 65.86 mg, respectively) as compared to negative control (31.54 mg).

### 3.2. Antacid Activity of RBAEDM

#### 3.2.1. Effect of Temperature Variation on RBAEDM pH

**Figure 2:**
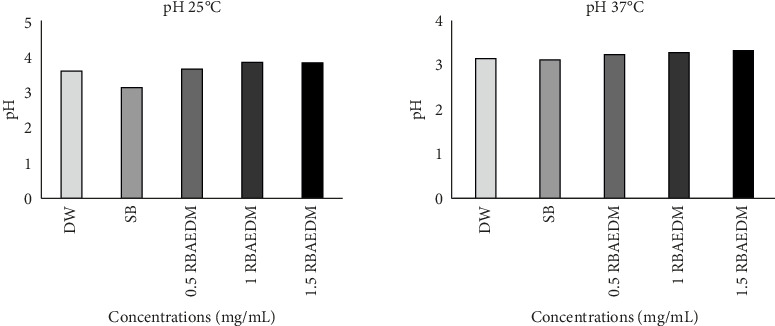
Effect of temperature variation on extract pH *N* = 5: number of animals per groups, values are expressed as mean ± standard error to the mean; DW, distilled water; SB, sodium bicarbonate; RBAEDM, root bark aqueous extract of *Diospyros mespiliformis* root bark extract.

At 25°C, the pH of the extract at different concentrations varied between 3.12 and 3.87, while at 37°C, it varied between 3.11 and 3.32 (Figure 2). However, the temperature had no impact on the pH.

#### 3.2.2. Neutralizing Effect of RBAEDM on Artificial Gastric Juice

The extract showed a concentration-dependent increase in the neutralizing effect on artificial juice; this effect was significant (*p* < 0.001) only shown at a concentration of 1.5 mg/mL as compared to distilled water (Table 2).

#### 3.2.3. Neutralizing Time of RBAEDM on Artificial Gastric Juice

A significant (*p* < 0.001) and concentration-dependent increase of neutralizing time (38, 44 and 62 min, respectively) of gastric acid compared with distilled water (24 min) is observed at the different concentrations of the extract (0.5, 1 and 1.5 mg/mL). This time was 46 min with sodium bicarbonate (Table 3).

### 3.3. Determination of the In Vitro Antacid Effect of RBAEDM

The extract showed a significant (*p* < 0.001) and concentration-dependent (0.5, 1 and 1.5 mg/mL) increase in the volume of artificial gastric juice consumed (3.8, 4.82 and 6.65 ± 0.30 mL, respectively) as compared with distilled water (2.65 mL). The increase in artificial gastric juice consumption was accompanied by a significant (*p* < 0.001) and concentration-dependent increase in H^+^ ion consumption (0.30, 0.30 0.41 mmol, respectively). As for sodium bicarbonate, the volume of juice consumed was 2.65 mL and the concentration of H^+^ consumed was 0.16 mmol (Table 4).

### 3.4. Antioxidant Effects of RBAEDM on Water Stress-Induced Ulcers

The effect of RBAEDM on some parameters of oxidative stress in water stress-induced rats is shown in Table 5. It is shown that the MDA level was significantly (*p* < 0.001) increased in the negative control as compared to that in the normal control. The administration of RBAEDM prior to cold water immersion of animals resulted in a significant (*p* < 0.001) decrease in the MDA level as compared with that of the negative control. The decrease in the MDA level was followed by a significant (*p* < 0.05; *p* < 0.01) increase in SOD and CAT activities when compared to that of the negative control. RBAEDM at 200 and 400 mg/kg resulted in a significant (*p* < 0.001) increase in nitrite levels (7.89, 11.41 mol/L) as compared with the negative control (5.66 mol/L).

### 3.5. Effects of *Diospyros mespiliformis* Aqueous Extract on Signs of Toxicity in Rats

**Figure 3:**
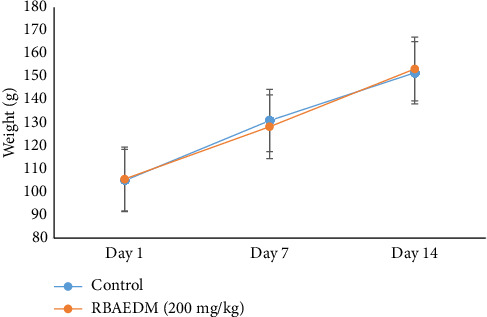
Effects of *Diospyros mespiliformis* aqueous extract on body weight.

The animals given a single dose of 2000 mg/kg of RBAEDM showed some signs similar to a toxic effect of the plant extract used. In fact, during the first 4 h following administration of the plant extract to the rats, a reduction in pain sensitivity and mobility was observed in the experimental rats. In addition, no deaths were observed during the study. These behavioural signs gradually returned to normal over the following 14 days of observation and did not significantly affect the relative weight of the experimental animals compared with that of the control (Figure 3). The extract did not cause the death of the animals throughout the experiment; the LD_50_ is therefore greater than 2000 mg/kg.

## 4. Discussion

Previous studies have shown the gastroprotective potential of the root bark aqueous extract of *Diospyros mespiliformis* on several gastric ulcer induction models [14]. The present study aimed to evaluate the antacid and cytoprotective effects of root bark aqueous extract of *Diospyros mespiliformis* on water immersion stress-induced gastric ulcers.

The pathophysiology of the stress-induced ulcer model involves, on one hand, the intraluminal increase of acid secretion by stimulation of the vagus nerve. This acid leads to the activation of pepsin, which is a proteolytic enzyme [28]. The combined action of acid and pepsin leads to an autodigestion of the gastric mucosa, which results in the destruction of the mucosa. Inhibition of acid secretion by antisecretory drugs or neutralization of gastric acidity by antacid drugs could prevent the formation of stress-related ulcers. Indeed, antacids cure ulcers by reducing gastric acid through neutralization, although they do not prevent acid secretion [8, 9]. In the present study, RBAEDM showed antacid potential by significantly increasing the neutralization of H^+^ ions from gastric juice. In terms of acid secretion inhibition, previous work has shown that RBAEDM decreases acid secretion via a mechanism involving the cholinergic and histaminic pathways [15]. RBAEDM could therefore act as both antacid and antisecretory agent.

Degradation of the gastric mucosa resulting from the corrosive action of acid can also lead to the production of free radicals (in endothelial cells and polymorphonuclear neutrophils), which also damage gastric mucosal tissue [2]. Several works have shown that substances or drugs that have the ability to scavenge free radicals via stimulation of antioxidant enzyme activity (CAT and SOD) could reduce lipid peroxidation [29] in gastric mucosal cells (characterized by the reduction of MDA production) and consequently gastric ulcer formation [30, 31]. In the present work, the significant inhibition of gastric ulcer formation observed in RBAEDM-treated animals could be justified in part by the significant (*p* < 0.001) decrease in MDA levels and the significant (*p* < 0.01, *p* < 0.001) increase in SOD and CAT activities. In addition, previous work on RBAEDM had revealed the presence of significant amounts of polyphenols, flavonoids that are classes of compounds with well-known antioxidant properties.

Stress-related gastric ulcers can also result from vasoconstriction of the gastric mucosa, causing a reduction in blood flow into gastric microcirculation (ischemia), with the consequent accumulation of deleterious products in gastric mucosa [32]. NO is an endogenous mediator that modulates both the repair and maintenance of gastric tissue integrity through its vasodilatory action, thus increasing blood flow to deliver nutrients and oxygen to the mucosa and clearing it from harmful substances [33, 34]. The results of our work showed a significant increase in nitrite (a stable metabolite of NO) levels in animals treated with RBAEDM. This could suggest a NO-mediated vasodilatory effect as a cytoprotective mechanism of RBAEDM.

The gastroprotective drugs used can also act on the secretion of mucus. Indeed, mucus plays a protective role by forming a continuous film on the epithelium surface that provides physical and chemical protection against acidity and enzymes of gastric juice [1]. In the present work, RBAEDM resulted in a dose-dependent increase of mucus secretion compared to the negative control. Our results are in line with those of [35] who showed that the gastroprotective effect of *Enantia chlorantha* would be related to the increase in mucus secretion.

The dose of a substance or its duration of exposure sometimes produces harmful effects on an organism. The use of medicinal plants is growing in most countries of the world because its use is mainly based on the idea that plants are a natural means of treatment devoid of any risk. Consumers often believe that natural is synonymous with harmless. However, a plant can be both useful and toxic. Acute toxicity studies have been carried out to determine the lethal dose 50 (LD_50_) of the aqueous extract of *Diospyros mespiliformis* root bark. The acute toxicity studies in this work showed that the administration of RBAEDM 2000 mg/kg reduced pain and mobility during the first 4 h. Adzu et al. [36] showed that *Diospyros mespiliformis* extract reduced pain. This suggests that the extract may have analgesic and muscle relaxant properties. The aqueous extract of *Diospyros mespiliformis* root bark is very slightly toxic, with an LD_50_ of over 2000 mg/kg. The aqueous extract of *Diospyros mespiliformis* can be classified in category 5 of very slightly toxic substances in the Globally Harmonised System of Classification of Toxic Substances [27].

## 5. Conclusion

RBAEDM could protect against gastric ulcer formation via the stimulation of mucus production, enhancement of antioxidant status, increase in blood flow and neutralization of H^+^ ions. Single-dose administration (2000 mg/kg) of the extract produced no toxic effects or mortality in rats.

## Figures and Tables

**Table 1:** Effects of RBAEDM on ulcerated surface, ulcer index, percentage inhibition and mucus mass.

Treatments	Dose (mg/kg)	Ulcerated surface (mm^2^)	% of ulcerated surface	Ulcer index	% of inhibition	Mucus mass (mg)
Normal control	—	—	—	—	—	47.14 ± 6.11
Negative control	—	37.60 ± 4.05	5.57	4.30 ± 0.24	—	31.54 ± 2.00
RBAEDM	100	28.40 ± 2.56	4.20	4.06 ± 0.41	5.58	38.42 ± 3.15
RBAEDM	200	15.00 ± 3.87^∗^	2.22	2.90 ± 0.77	32.55	41.82 ± 3.42
RBAEDM	400	9.20 ± 3.81^∗∗^	1.36	1.70 ± 0.44^∗^	60.46	70.64 ± 7.28^∗∗∗^
Verapamil	50	34.40 ± 8.61	5.09	3.06 ± 0.79	28.83	65.86 ± 4.76^∗∗∗^

*Note: N* = 5: number of animals per groups; values are expressed as mean ± standard error to the mean.

Abbreviation: RBAEDM, root bark aqueous extract of *Diospyros mespiliformis*.

^∗^ *p* < 0.05, ^∗∗^*p* < 0.01, ^∗∗∗^*p* < 0.001: significant difference compared to the negative control.

**Table 2:** Neutralizing effect of RBAEDM on artificial gastric juice.

Treatments	Concentration (mg/mL)	pH
Distilled water	—	3.74 ± 0.06
RBAEDM	0.50	3.89 ± 0.03
RBAEDM	1.00	4.18 ± 0.07
RBAEDM	1.50	5.16 ± 0.30^∗∗∗^
Sodium bicarbonate	1.00	5.38 ± 0.07

*Note: N* = 5: number of animals per groups; values are expressed as mean ± standard error to the mean.

Abbreviation: RBAEDM, root bark aqueous extract of *Diospyros mespiliformis*.

^∗∗∗^ *p* < 0.001: significant difference compared to the negative control.

**Table 3:** Neutralizing time of RBAEDM on artificial gastric juice.

Treatments	Concentration (mg/mL)	Time (min)
Distilled water	—	24.50 ± 0.76
RBAEDM	0.50	38.39 ± 1.90^∗∗∗^
RBAEDM	1.00	44.50 ± 0.91^∗∗∗^
RBAEDM	1.50	62.28 ± 0.91^∗∗∗^
Sodium bicarbonate	1.00	45.99 ± 2.95^∗∗∗^

*Note: N* = 5: number of animals per groups; values are expressed as mean ± standard error to the mean.

Abbreviation: RBAEDM, root bark aqueous extract of *Diospyros mespiliformis*.

^∗∗∗^ *p* < 0.001: significant difference compared to the negative control.

**Table 4:** Determination of the antacid capacity of RBAEDM.

Treatments	Concentration (mg/mL)	VAGJC (mL)	mmol of H^+^
Distilled water	—	2.65 ± 0.06	0.16 ± 0.00
RBAEDM	0.50	3.80 ± 0.03^∗∗∗^	0.23 ± 0.00^∗∗∗^
RBAEDM	1.00	4.82 ± 0.09^∗∗∗^	0.30 ± 0.00^∗∗∗^
RBAEDM	1.50	6.65 ± 0.30^∗∗∗^	0.41 ± 0.01^∗∗∗^
Sodium bicarbonate	1.00	12.00 ± 0.31^∗∗∗^	0.75 ± 0.01^∗∗∗^

*Note: N* = 5: number of animals per groups; values are expressed as mean ± standard error to the mean.

Abbreviations: RBAEDM, root bark aqueous extract of *Diospyros mespiliformis*; VAGJC, volume of artificial gastric juice consumed.

^∗∗∗^ *p* < 0.001: significant difference compared to the negative control.

**Table 5:** Effect of RBAEDM on some parameters of oxidative stress and nitrites on water immersion stress.

Treatments	Dose (mg/kg)	MDA (μmol/mg protein)	SOD (U/mg protein)	CAT (μmol H_2_O_2_/min/mg protein)	GSH (mmol/g protein)	Nitrites (mol/L)
Normal control	—	43.69 ± 0.08	125.55 ± 5.00	24.61 ± 1.05	78.22 ± 9.32	6.02 ± 3.32
Negative control	—	102.58 ± 7.09^###^	123.87 ± 4.15	16.73 ± 0.78^##^	77.59 ± 14.62	5.66 ± 2.35
RBAEDM	100	39.42 ± 5.94^∗∗∗^	141.56 ± 3.23^∗^	21.14 ± 1.38^∗^	110.31 ± 10.26	6.10 ± 3.17
RBAEDM	200	39.80 ± 7.02^∗∗∗^	150.87 ± 2.00^∗∗^	25.01 ± 1.98^∗∗^	81.50 ± 10.24	7.89 ± 3.04^∗∗∗^
RBAEDM	400	36.65 ± 5.71^∗∗∗^	188.49 ± 6.26^∗∗∗^	25.20 ± 1.17^∗∗^	92.42 ± 6.78	11.41 ± 2.55^∗∗∗^
Verapamil	50	25.52 ± 0.04^∗∗∗^	151.59 ± 7.01^∗∗^	25.53 ± 1.86^∗∗^	96.56 ± 8.43	6.21 ± 2.20

*Note: N* = 5: number of animals per groups; values are expressed as mean ± standard error of the mean, MDA: malondialdehyde, CAT: catalase, GSH: reduced glutathione.

Abbreviations: DMRBAE, *Diospyros mespiliformis* root bark aqueous extract; SOD, superoxide dismutase.

^∗^ *p* < 0.05, ^∗∗^*p* < 0.01, ^∗∗∗^*p* < 0.001, significant difference compared to negative control.

^##^ *p* < 0.01, ^###^*p* < 0.001, significant difference compared to normal control.

## Data Availability

All data from this work are available from the corresponding author on reasonable request.
